# Comparison of Mechanical Behavior of Clear Aligner and Rapid Palatal Expander on Transverse Plane: An In Vitro Study

**DOI:** 10.3390/bioengineering11020103

**Published:** 2024-01-23

**Authors:** Alessandro Bruni, Andrea Abate, Cinzia Maspero, Tommaso Castroflorio

**Affiliations:** 1 Surgical, Medical and Dental Department, University of Modena and Reggio Emilia, 41124 Modena, Italy; halex.bruni@gmail.com; 2 Department of Sciences Integrated Surgical and Diagnostic, University of Genova, 16126 Genova, Italy; andreabate93@gmail.com; 3 Department of Biomedical Surgical and Dental Sciences, University of Milan, 20142 Milan, Italy; 4 Fondazione IRCCS Cà Granda, Ospedale Maggiore Policlinico, 20122 Milan, Italy; 5 Private Practice, 10129 Torino, Italy; tommaso.castroflorio@gmail.com

**Keywords:** maxillary expansion, clear aligner, malocclusion

## Abstract

(1) Background: This study aims to investigate, within a controlled laboratory environment, the magnitude of the transversal load and the force decay over time produced by clear aligners in comparison to a Rapid Palatal Expander (RPE). (2) Methods: Resin models of a dental maxillary arch, additively manufactured from an intraoral scan, were inserted in a testing machine with uniaxial load cells to measure the force trend over time expressed by RPE and clear aligners. The mechanical load was recorded during a certain timeframe for both appliances. (3) Results: The force expressed by the RPE ranged from 30 to 50 N for each activation, decreasing with a nonlinear pattern over time. The force expressed by the clear aligner ranged from 3 to 5 N, decreasing with a linear pattern over time. In contrast, the force generated by the clear aligner fell within the range of 3 to 5 N, showing a linear reduction in force magnitude over the observed period of time. (4) Conclusions: The RPE exerted a force magnitude approximately ten times greater than that generated by clear aligners. Nevertheless, it is essential to acknowledge that the oral environment can significantly influence these results. These limitations underscore the need for caution when applying these findings to clinical settings.

## 1. Introduction

Transverse maxillary deficiency represents one of the most pervasive problems in the craniofacial region, with several clinical features occurring in all the planes of space. These features could appear independently but, in most cases, they take place together, as in what might be termed *maxillary deficiency syndrome* [1]. The etiology of transverse maxillary deficiency is multifactorial. According to the literature, it appears to be confirmed that transverse discrepancy between the maxilla and mandible may have a genetic–hereditary basis [2]. Also, cleft palate [3] and other malformations of the head and neck (e.g., Marfan syndrome [4], Klippel–Feil syndrome [5], Treacher Collins syndrome [6], craniosynostosis [7,8,9]) could lead to a decreased transverse dimension of the upper jaw.

Narrow maxilla occasionally derives from neonatal intubation resulting in trauma [10] or from prolonged pressure on the palate such as via early weaning and associated low-impact muscular activity from bottle feeding [11].

Habits including non-nutritive sucking [11], adaptive swallowing behavior [12], open mouth posture connected to predominant mouth breathing, excessive use of dummies and baby bottles [11], and low tongue position [13] represent common causes of narrow maxilla. However, some authors have found no association between prolonged sucking habits or mouth breathing with a transverse maxillary deficiency [13].

Additional causes include conditions associated with decreased tonic muscle activity [13], myopathies (e.g., myotonic dystrophy (MyD) and Duchenne muscular dystrophy (DMD) [14]), scarring as a result of post-traumatic injury (e.g., burns), functional shifts to achieve maximal intercuspation, juvenile rheumatoid arthritis, and unilateral condylar hypoplasia or hyperplasia [15].

Maxillary transverse deficiency usually requires expansion of the palate, achievable through several treatment modalities [16] that practitioners select based on scientific evidence [17] together with their personal beliefs and experiences [18]. The first concept of maxillary expander dates back to 1860 [19], when E. H. Angell published the first case report related to an appliance that used two contra-rotating screws to acquire enough space for maxillary canines.

The non-spring-loaded jackscrew appliances act by transferring the mechanical load across the mid-palatal suture, promoting the disjunction of the upper jawbone when interdigitation and bony bridging are still incomplete, modulating bone remodeling and formation [20,21].

Maxillary expansion can be rapid (RME) or slow (SME) depending on the activation protocol of the active part of the appliance (i.e., number of screw turns per day).

Alternatively, it can be achieved with calibrated and continuous forces that promote maxillary expansion using a Ni-Ti leaf springs palatal expander (Leaf Expander) that has a small-sized body and is similar to a conventional Hyrax expander [22,23,24]. Several studies have endorsed the utilization of devices that facilitate gradual expansion to minimize unwanted side effects [25,26,27] and enhance long-term stability following expansion [28,29].

The *Rapid Palatal Expander* (RPE) is one of the most used appliances in cases of upper jaw constriction because of its versatility in achieving skeletal expansion [30] and dentoalveolar expansion [31] (or both), its ease of use, and its broad acceptance by the scientific community [32]. The RPE acts by transferring the forces produced by the activation of a jackscrew to the anchorage unit (first permanent molars or second primary molars in the case of tooth-borne appliances; TADs in the case of bone-borne appliances; teeth and TADs in case of hybrid tooth–bone-borne appliances) and then channeling that force to the midpalatal and circummaxillary sutures [33], causing the separation of the two maxillary bone halves [33]. The progressive increase in resistance from surrounding structures results in dentoalveolar transverse expansion that exceeds the skeletal expansion [34,35,36,37], except when the anchor units are skeletal rather than dental [38].

Several studies investigated the biomechanical behavior of jackscrew appliances through finite elements analysis [39,40,41,42] or in both in vitro [39,43,44,45] and in vivo settings [46,47,48,49,50]. However, the underlying biological processes behind maxillary skeletal expansion remain inadequately understood, and the clinical parameters for predicting expansion success are still lacking.

Recent studies showed that *clear aligners* could achieve satisfactory maxillary arch development in adults [51,52,53,54,55,56] and growing patients [57,58,59,60,61], but despite the significant surge in research activities and the growing number of publications on CAT which have been observed in recent years [62], the mechanical features of clear aligners during expansion have not yet been tested. It has been demonstrated that the expansion achieved using CAT is predominantly dentoalveolar, achieved mainly through the tipping movement of the posterior teeth, with the tilt angle increasing as the arch expands [53]. Given that the force exerted by clear aligners is not yet clearly understood, it is crucial to investigate this subject further to gain a deeper understanding of the biomechanics involved.

Thus, the aim of the current investigation was to test, in a laboratory setting, the magnitude of the transversal load and the force decay over time produced by clear aligners compared with RPE.

## 2. Materials and Methods

The study protocol received approval from the Institutional Ethics Committee (Città della Salute e della Scienza di Torino, approval number: 0006323) at the coordinating center. The study was conducted in accordance with the Declaration of Helsinki, adhering to relevant national and international regulatory requirements.

**Figure 1:**
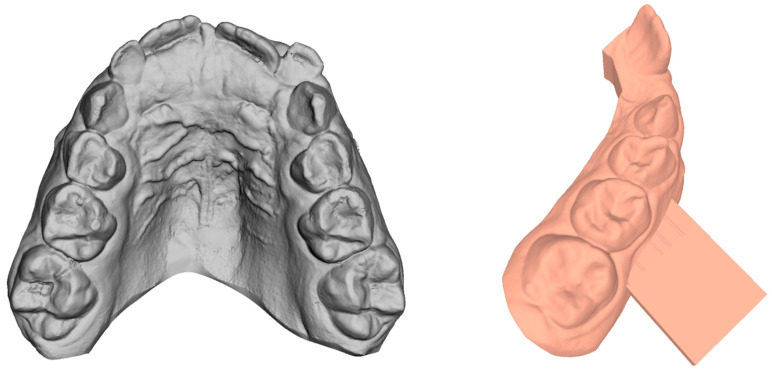
Digital dental cast obtained from intraoral scan and CAD project of the solid fixture arm for testing machine gripping.

In the present study, experimental models reproducing the maxillary dental arch were used to test the mechanical behavior of the clear aligner and RPE. The models were additively manufactured using a stereolithography (SLA) 3D printer (Form 2, FormLabs, Somerville, MA, USA) with 50 μm resolution, with dental model resin (Formlabs, Somerville, MA, USA) as the printing material. SLA printers use a moving ultraviolet (UV) laser beam to selectively cure/solidify photopolymer patterns layer by layer. The models were printed from three-dimensional digital dental casts (Figure 1) obtained using a laser scanner with a reported manufacturing accuracy of 20 μm (iTero; Align Technologies, San Jose, CA, USA). Both resin dental models were manufactured with two parallels to the transversal plane rectangular solid fixture arms (12 mm length, 12 mm width, 3 mm height; Figure 1), designed with CAD software (3Shape Ortho System™, 3Shape, Copenhagen, Denmark), to be inserted in a testing machine (Bose ElectroForce^®^ Planar Biaxial Test Bench, TA Instruments, New Castle, DE, USA) with a 225 N uniaxial load cell.

The force trend over time exerted along the mediolateral direction was measured through a 225 N uniaxial load cell for both appliances:

1. Invisalign^®^ First Phase I treatment (Align Technology, Inc., Santa Clara, CA, USA)

The Invisalign^®^ First clear aligners are fabricated in a multilayer aromatic thermoplastic polyurethane/co-polyester, 0.75 mm (0.030″) thick, with a fine 3D manufacturing process. A series of optimized and conventional attachments were used to improve the retention at the model/appliance interface and to perform the desired tooth displacement. The ClinCheck^®^ Pro software (Align Technology, Inc., Santa Clara, CA, USA) was used to plan orthodontic movements. Concerning the staging, permanent molars moved buccally first, using the rest of the arch as anchorage. Because of the short clinical crowns of deciduous teeth, specific attachment shapes were designed to increase aligner retention and to control the tipping movement (in order to obtain torque compensation).

2. Tooth-borne Hyrax-type maxillary expander

The Hyrax-type maxillary expander is fixed to the upper second deciduous molars of the resin model using laser-melted clasps, modeled surrounding the molars, connected via a framework of 0.9 mm stainless steel wires, laser-welded to a midline 10 mm self-locking screw (A2620 rapid expander; Leone orthodontic products, Sesto Fiorentino, Firenze, Italy; 0.8 mm, complete turn—4 activations). The framework is soldered to the metal clasp and extends on the palatal side to the deciduous canines on the resin model. The expander will be fabricated by a qualified laboratory technician (Novadental Lab, Torino, Italy).

**Figure 2:**
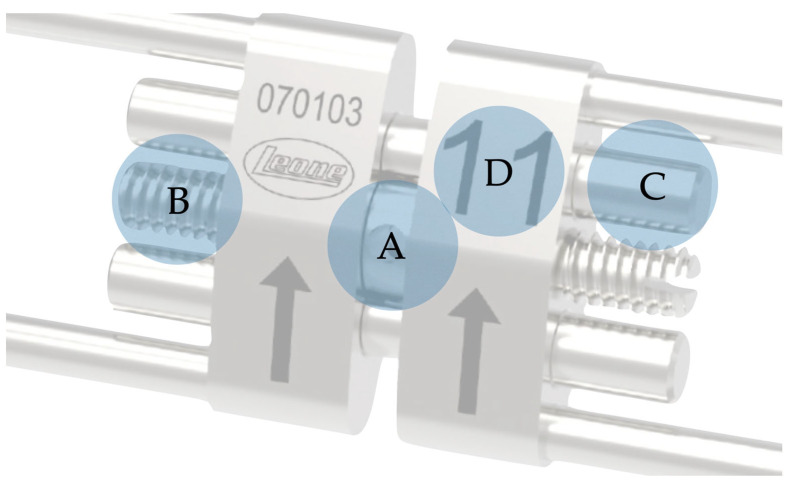
Components of the basic jackscrew: (**A**) perforated cylinder; (**B**) leadscrew; (**C**) guide pins; (**D**) platform.

The jackscrew comprises four primary components (Figure 2):

(1) Perforated Cylinder: The perforated cylinder, encircling the leadscrew, is rotated to induce expansion. Featuring four holes, each turn of the key corresponds to a quarter turn of the cylinder. Each activation corresponds to 0.2 mm of expansion; thus, completing a full cycle of four turns results in a total expansion of 0.8 mm.
(2) Leadscrew: The length of the leadscrew dictates the maximum achievable expansion. Jackscrews vary between 3 and 18 mm of maximum expansion (removable expanders incorporate smaller jackscrews, achieving less expansion, typically ranging from 3 to 7 mm). The selection of leadscrew length is based on the patient’s transverse discrepancy.
(3) Guide Pins: Strategically positioned above and below the leadscrew, guide pins offer structural support. Early iterations of modern jackscrews lacked a guidance mechanism by which to stabilize the appliance against torsion. A standard four-leg RPE utilizes two guide pins, while a two-leg RPE requires only one guide pin. These mono-guided jackscrews are commonly referred to as mini-jackscrews due to their smaller platform.
(4) Platform: Housing the leadscrew and guide pins, the platform serves as a linkage to the framework. Its surface is marked with two identifiers: a small arrow denoting the direction of turning (opening) with the key; and a number, known as the nominal size, indicating the approximate maximum expansion amount in millimeters.

The appliance’s main structure will be sintered with Remanium^®^ Star CL (Dentaurum, Ispringen, Germany), a metal alloy powder, after computer design with a dedicated software (3Shape Ortho System™, 3Shape, Copenhagen, Denmark). The stereolithographic files were sent to a laser-melting center (Cadent Srl, Augsburg, Germany), where the RPE was manufactured. This additive production builds the appliance layer by layer with the above-mentioned powder, which is locally melted with laser. After cooling down, the raw appliance has to be cleaned of the not-melted powder and then polished. The expansion jackscrew was added with laser-welding, and the bonding sites have to be sandblasted for an optimized interface with the bonding material.

The RPE was bonded to the resin model with an orthodontic composite (Transbond XT, 3M Unitek, Monrovia, CA, USA), light-cured by the means of a halogen lamp (Optilux, Kerr, Orange, CA, USA) for 20 s per tooth.

**Figure 3:**
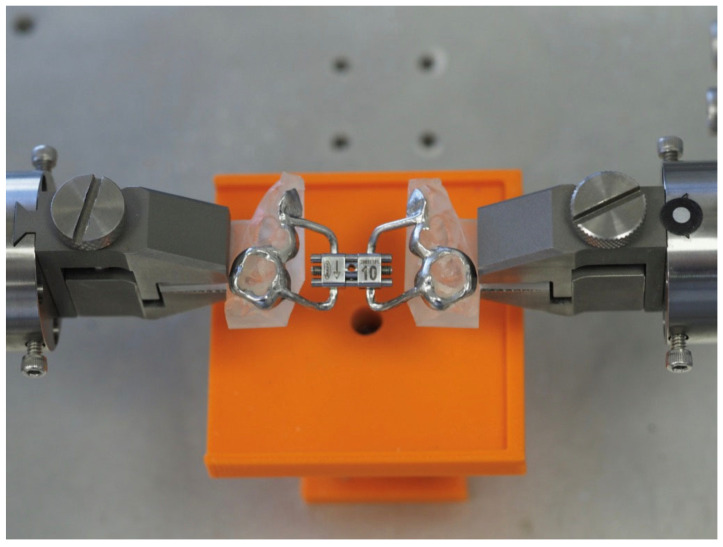
Resin model with RPE placed in the testing machine.

**Figure 4:**
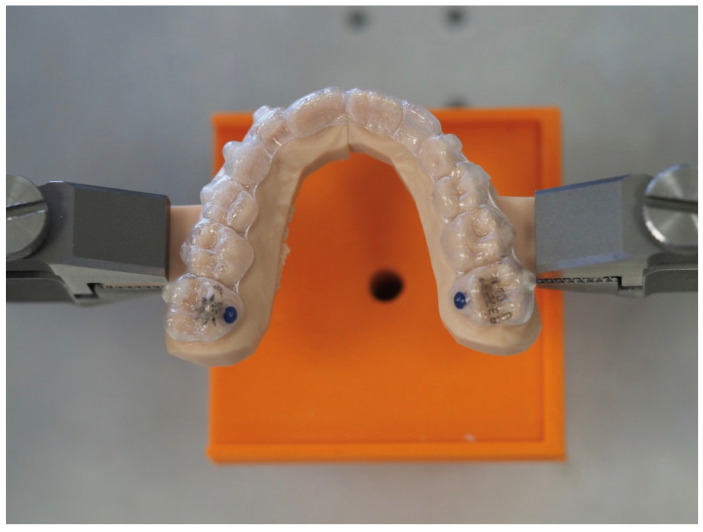
Resin model with CA placed in the testing machine.

The resin model with the corresponding appliance was placed in the testing machine (Bose ElectroForce^®^ Planar Biaxial Test Bench, TA Instruments, New Castle, DE, USA) by grabbing the rectangular solid fixture arms, trying to keep the expander as aligned as possible on the horizontal plane, as shown in Figure 3 and Figure 4. The specimens were clamped by titanium machine grips that were specifically developed for biomaterials and have knurled-flat faces to prevent slipping. The analysis of the video recordings demonstrates that there were neither anomalous behaviors nor failures near the clamps. Sliding through the testing grips was excluded, too, as no abrupt increase or decrease was detected in the experimental curves. No marks were observed on the specimen ends, and the extension of the grasped ends was found to be unchanged.

The testing room temperature was 20 °C, while the humidity ranged between 40% and 65%. The displacement was set as equal to zero when a 0.05 N force was recorded.

Once the devices were positioned, the resulting compression force was recorded after activation (in the case of RPE) or appliance placing (in the case of the clear aligner) with a sampling frequency of 1 Hz for RPE and one point every 30 min for the aligner.

The activations of RPE were performed, with a specific timeframe pattern, by means of a stainless steel key with a diameter of 1 mm. The key was inserted fully into the hole of the screw to perform the turns.

The activation pattern for the RPE was as follows:

− Semi-rapid: one activation (one quarter turn; 0.2 mm) every 24 h.

The force magnitude of clear aligners was measured over a 12 h timeframe, taking into account the maximum duration of retention within the oral cavity before the removal for meals.

The force trends over time were exported in MATLAB 2020a (MathWorks, Natick, MA, USA). A polynomial of degree 10 was fitted on the experimental data collected during the test performed with the clear aligner. Regarding the RPE, a moving averaging filter was used to smooth the signal, although the peak force values were maintained.

The graphs obtained represent the averages of three successive measurements to minimize potential errors.

## 3. Results

The analysis of video recordings demonstrated that there were neither anomalous behaviors nor failures near clamps; therefore, all acquired data have been elaborated.

**Figure 5:**
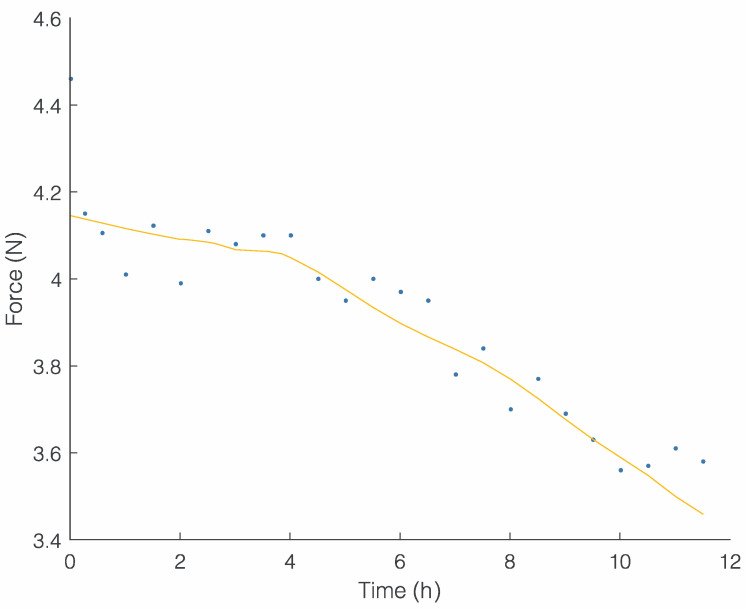
Force (N) trend over time (h) of clear aligner.

**Figure 6:**
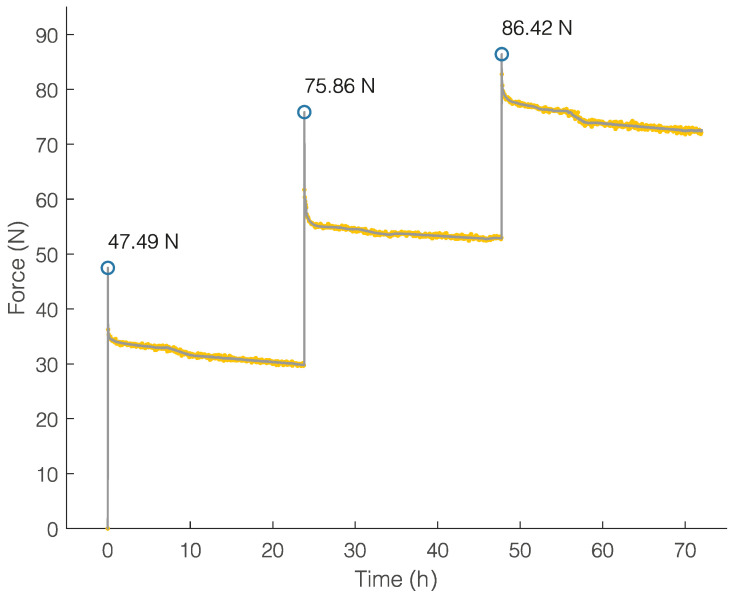
Force (N) trend over time (h) of RPE.

The mechanical load measurements along the mediolateral direction were plotted as a function of time for both appliances (clear aligner, Figure 5; RPE, Figure 6). The clear aligner exhibited an almost linear decay in overall force trend after approximately three hours, starting at an initial force of around 4.2 N and gradually decreasing to approximately 3.4 N after 12 h (Figure 5).

The RPE exhibited a rise in force values and a sharp decrease in the correspondence of each activation, and a slow decay was recorded during the 24 h between consecutive activations. The forces generated after three activations were significantly higher than those generated after one activation; the force increases immediately after the three activations were 47.49 N, 46.06 N, and 33.59 N, respectively, while the force peak values are depicted in Figure 6.

Significantly higher force levels were observed with the Rapid Palatal Expander (RPE) compared to those exhibited by clear aligners.

## 4. Discussion

This study analyzed the forces produced by two different appliances for treating transverse maxillary deficiency in an in vitro setting.

The RPE is still the most widely used appliance in facing this common feature of many malocclusions, but the scientific evidence about its mechanical behavior is still lacking. Indeed, despite the extensive number of articles investigating the clinical short- [63,64,65] and long-term [32,65] effects of RPE, the literature surrounding the amount of forces produced by this appliance is still limited [39,43,46,49,50,66].

The rapid rise in force levels immediately after each activation with a non-linear decay pattern measured in the present study is consistent with previous findings [46,49,50]. However, the former attempts to identify the force levels expressed when activating the RPE jackscrew were made in different experimental settings [46,49,50]. Despite the different methods used by the authors [46,49,50], a similar pattern of force trend over time was assessed.

The activation patterns tested in the present study are in accordance with the most-common protocols [67]. Also, the cumulative force levels detected after repeated activation of the central expansion screw were in accordance with the results of the previous studies [49,50], and they are sufficient to guarantee the separation of the median palatine suture in pre-adolescent and adolescent patients, akin to almost all the different types of expanders tested in the literature [45]. The resulting stress after the appliance’s activations is distributed in the maxilla as well in the neighboring skull bone [41,42]. The biological response behind skeletal expansion occurs when the force applied to the teeth and the maxillary alveolar processes exceeds the limits needed for orthodontic tooth movement, causing the separation of the two maxillary bone halves [33].

The maturation of the circummaxillary sutures, according to several studies [40,48,68,69], accounts for a significant part of adult resistance to midpalatal suture separation. The resistance to force encountered during palatal expansion is also dependent on the material properties of bones, which remain constant over time, as well as on bone mass, which changes with age or sex [70]. It was assumed that soft-tissue pressure to the upper arch also contributes to resistance to expansion, influencing its long-term effects (0.6 g/mm^2^ per mm of expansion) [71].

Thermoplastic appliances have a long-standing history in orthodontics. According to the literature, the first attempt to treat minor malocclusion using a clear, vacuum-formed tooth-positioning appliance can be dated back to 1945 [72]. The primordial technique, consisting in a complex and time-consuming procedure, precluded its broad diffusion until the arrival of certain technological innovations and the introduction of new materials which allowed for the evolution of CAT as a feasible alternative for treating several malocclusions [73].

The use of clear aligners has the potential to provide a more esthetic and comfortable treatment, as well as better oral hygiene maintenance [74]. Customized virtual diagnosis and treatment and biomechanical planning also have the potential to decrease complications, increase treatment efficiency, and enhance treatment outcomes [75].

The potential benefits of CAT are tempered by the uncertainty surrounding its capability to perform; the latest scientific evidence suggests a reduced efficacy in controlling several types of orthodontic tooth movements (OTMs) compared with fixed orthodontic appliances [73,76,77,78], which has reduced the wide acceptance of the technique within the scientific community.

Apart from these limitations, the popularity of CAT has been growing steadily, as observed by the relevant rise in market share in recent years [79]. This has sparked academic interest and has resulted in a significant surge in research activities [62].

With maxillary transverse deficiency being one of the most encountered clinical conditions in daily orthodontic practice, it is therefore important to establish whether the CAT could be a feasible approach to facing it.

To our knowledge, there exist no studies investigating force trend over time exerted along the transversal direction by clear aligners in a laboratory setting.

Divergently from the RPE, the clear aligner’s mechanical load detection showed a moderate rise in force levels with a linear decay pattern. The magnitude of the force measured was similar to the values obtained for other appliances used for dental–alveolar expansion [80]. The moderate magnitude of force (below 5 N) has been proved to be sufficient to obtain a skeletal widening of the maxilla in CLP Patients [81,82], while it could be insufficient to separate a progressively maturing suture if a cleft jaw and palate are not present [81]. The vast majority of publications have reported a higher sutural separation rate (40–58%) obtained through RPE [83,84,85,86] compared to slow palatal expanders (16–40%) [87,88,89,90,91]. However, some animal studies on SPE have demonstrated sutural separation comparable to those of RPE [87,92]. The main effects of SPEs are dentoalveolar (molars buccal tipping and mesiobuccal rotation); an average mesiobuccal rotation of the molars of 26° [93] and 2° [94] to 24° [87] of buccal molar tipping has been reported with the use of SPEs compared to the minimal buccal molar tipping observed when using RPE [95]. It has been widely demonstrated that using clear aligners, the expansion is achieved more by dental tipping than by bodily translation [52,53,96,97,98]. This is why forces act at a distance from the molar’s center of resistance, causing buccal tipping of the posterior segment, which is frequently detrimental to the treatment objective. Furthermore, the consequent pervasive sliding effect between the plastic and tooth crown, along with the relatively low stiffness that leads to uncontrolled tipping during expansion, will cause the aligner to flare, losing control as dissociation between the tooth and plastic occurs [99]. The use of attachments on the buccal or lingual surface of the posterior teeth helps improve third-order control by counteracting the undesired tipping moment generated by a couple with opposite forces acting at the occlusal surface and at the gingival aspect of the attachment [99,100].

Results from the present study place the CAT alongside the SPEs, with the potential to exert a force magnitude below 5 N. Although there are similar magnitudes, there are several differences between the force trend over time in the different types of SPE appliances. The tooth-borne jackscrew maxillary expanders, activated with a rate of 0.5 mm per week [101], express intermittent forces that tend to decline to zero until the next activation [67]. Fixed appliances like Quad-Helix or the Nitanium Palatal Expander express continuous forces, are lighter, and are theoretically considered the most effective in producing tooth-movements [67]. Unlike traditional expanders, the Leaf Expander employs a flexible leaf spring mechanism that exerts controlled forces on the maxillary arch. The key advantage lies in its ability to distribute forces more evenly, reducing the risk of undesirable side effects. The literature indicates that the Leaf Expander, in comparison to the traditional RME, induces less discomfort while achieving a comparable degree of expansion [22,23,24]. A recent three-dimensional study validated the efficacy of both the Leaf Expander and RME in addressing maxillary deficiencies among patients with mixed dentition. In terms of skeletal expansion, only two parameters exhibited statistically significant differences favoring the RME group [22]. However, these differences, measuring less than 1 mm, may be deemed clinically irrelevant. In terms of considered dentoalveolar variables, no statistically significant distinctions were observed between the Leaf Expander and the conventional RME. These findings suggest that the Leaf Expander serves as a compliance-free and viable alternative to RME in maxillary expansion therapy [22].

Clear aligners, as with all the removable appliances, express interrupted forces that decline abruptly to zero when the appliance is removed by the patients [67], so their effect is strongly dependent on patient compliance.

Although the methods used in the present study allow for the evaluation of detailed behavior of different appliances, the testing machine measures the expansion force along the transverse axis only, although it has been demonstrated that appliances generate forces in all three spatial dimensions [46]. Another important bias is represented by the laboratory settings in which the forces were transferred to a resin model connected to the sensors, differing significantly to the appliance–teeth interface existing in the human mouth. The mechanical properties of thermoplastic materials used for clear aligner manufacturing strongly depend on the material types and environmental conditions in which they are used [102].

In the oral environment, both temperature and moisture significantly accelerate the stress relaxation of orthodontic thermoplastic appliances, causing the deterioration of their mechanical properties over time without significant differences between various materials [103]. For all these reasons, predicting orthodontic forces expressed by clear aligners and, consequently, the relative tooth movement, became much more challenging. Further studies investigating the in vivo behavior of clear aligners are necessary in order to clarify the potential of the techniques for treating several types of malocclusions.

As a suggestion for future research, it would be valuable to include the Leaf Expander in the comparative analysis or to explore direct printed aligners that utilize shape-memory materials [104,105], which may express higher forces with the application of a specific stimulus.

## 5. Conclusions

Taken together, the measurements obtained from the experiment suggest the following:

− The cumulative magnitude of the transversal load in response to RPE activations ranges between 30 and 50 N for each activation. An immediate decrease in the mechanical load is noticeable after each activation, followed by a progressive and gradual reduction over time.
− The magnitude of the transversal load at the clear aligner placement ranges between 3 and 4 N and declines to zero any time the appliance is removed.
− The mechanical behavior of both appliances could vary significatively in the oral environment.

Although the use of clear aligners in dentistry is growing, there is plenty of room for improvement in terms of the predictability of certain tooth movements and for further development. Since research activities have strengthened in recent years, substantial advances can be expected soon.

## Data Availability

The data underlying this article will be shared on reasonable request to the corresponding author.
